# Inhibition of *Aeromonas hydrophila* and intestinal pathogenic bacteria in Nile tilapia (*Oreochromis niloticus*) fish by silver nanoparticles

**DOI:** 10.5455/javar.2025.l928

**Published:** 2025-06-11

**Authors:** Samyah D. Jastaniah, Najah M. Albaqami

**Affiliations:** 1 Department of Biological Sciences, Faculty of Sciences, King Abdulaziz University, Jeddah, Saudi Arabia

**Keywords:** Coated AgNPs, antibacterial activity, pathogenic bacteria, motile *Aeromonas septicemia*, Nile tilapia

## Abstract

**Objective:**

This research investigated the antibacterial properties of coated silver nanoparticles (CAgNPs) and non-coated silver nanoparticles (NCAgNPs) against bacterial pathogens relevant to Nile tilapia (*Oreochromis niloticus*), using both *in vitro* and *in vivo* methods.

**Materials and Methods:**

The antibacterial activity of CAgNPs and NCAgNPs was evaluated *in vitro* against seven bacterial pathogens: *Staphylococcus aureus*, *Bacillus cereus*, *Escherichia coli*, *Aeromonas hydrophila*, *Listeria monocytogenes*, *Salmonella enterica*, and *Aeromonas sobria*. For the *in vivo* assessment, 200 Nile tilapia were divided into five treatment groups. The control treatment group was fed a standard diet, but the four treatment groups were fed the standard diet mixed with either 100 or 200 mg/kg of CAgNPs or NCAgNPs, respectively, and the growth indices, antioxidant parameters, immune functions, and intestinal microbiota were assessed. When the experiment was finished, 20 fish of every group were infected with *A. hydrophila*.

**Results:**

*In vitro* assays demonstrated significant antibacterial activity of both CAgNPs and NCAgNPs at 200 µg/ml (p < 0.05) against every bacterial strain that was studied, with moderate activity observed at 100 µg/ml. CAgNPs exhibited larger inhibition zones (30.0 ± 0.58 mm to 36.33 ± 0.88 mm) compared to NCAgNPs, with a minimum inhibitory concentration of 150 µg/ ml. Dietary supplementation with 200 mg/kg CAgNPs improved growth performance, enhanced immune parameters, reduced oxidative stress, and decreased intestinal bacterial load in Nile tilapia. Furthermore, dietary supplementation with 100 mg/kg CAgNPs was more efficient than 100 mg/kg NCAgNPs in reducing intestinal bacterial colonization in fish.

**Conclusion:**

These findings indicate the possibility of CAgNPs as an efficient antimicrobial factor in aquaculture. CAgNPs may offer a promising alternative to conventional antibiotics by improving tilapia health and antioxidant status, enhancing immune function, and increasing disease resistance.

## Introduction

A quickly growing area of the food economy is aquaculture, providing a reliable origin of seafood and mitigating pressure on wild fish stocks. It plays an important role in providing global seafood demand while also helping environmental conservation. However, the aquaculture production faces significant obstacles due to bacterial and other pathogenic infections [1].

Tilapia is a commercially important fish species because of its rapid growth, consumer acceptance, and resilience, making it amenable to aquaculture. It represents a valuable and affordable protein source for a growing global population [2]. Furthermore, tilapia farming can contribute to economic development in many regions by generating employment and income for local communities [3,4]. During cultivation, this species is exposed to several infectious diseases caused by bacteria, viruses, or fungi. These diseases can significantly impact production by increasing mortality rates and leading to economic losses [5]. The *Aeromonas hydrophila* species is a freshwater bacterium that can cause septicemia and gastroenteritis in fish, leading to significant disease outbreaks [3]. Moreover, *A*. *hydrophila* is commonly found in water and can exhibit various virulence factors that increase its ability to cause disease [6]. These factors are influenced by environmental conditions such as temperature and pH, which are important for the bacteria’s survival, growth, and pathogenicity [1].

Further research investigating the physicochemical traits, stability, efficacy, and distribution of nanoparticle complexes for fish disease management is essential. A key challenge in fish farming is mitigating potential nanoparticle toxicity while ensuring long-term immunity [7–9]. Continued fundamental study is essential to advance fish immunization strategies and enhance the efficacy of nanotechnology-based vaccines [10].

Although the green composition of silver nanoparticles (AgNPs) has been indicated to exhibit toxic effects at certain concentrations [11], AgNPs have the potential for controlling bacterial fish pathogens and preventing disease outbreaks in aquaculture [12]. For instance, studies have shown that fish exposed to 0.8 mg/l AgNPs did not exhibit adverse health effects following *A*. *hydrophila* infection [12].

Therefore, utilizing, for example, *Moringa oleifera* extract in AgNP synthesis could offer a significant advantage for disease management in aquaculture [11]. However, the development of standardized application protocols and the phasing out of antibiotic growth promoters are essential to the efficient and safe application of AgNPs as nutritional supplements. Several experiments have evaluated the antibacterial impact of AgNPs toward fish pathogens, as well as their potential toxicity in species such as Nile tilapia [1], rainbow trout species (*Oncorhynchus mykiss*) [3], and common carp fish (*Cyprinus carpio*) [11].

AgNPs are considered an environmentally friendly and low-cost replacement to traditional antimicrobials for controlling *A*. *hydrophila* infections in fish, offering potential benefits as antiseptics or antimicrobial agents for fish health management. AgNPs have been synthesized using extracts from various plant sources, including *Cedrus deodar* leaves [13,14], *Carica papaya*, *Eucalyptus terticornis*, *Mangifera indica*, *Musa paradisiaca* [15,16], and *M. oleifera* [11,17,18].

Conflicting results exist in the previous studies regarding the impacts of AgNPs on aquaculture health and growth, with some studies reporting adverse effects while others suggest potential benefits [1,3,10,11]. The application of coated AgNPs to mitigate *A*. *hydrophila* strong pathogen and the influence of environmental elements represents a promising strategy for disease prevention and control in aquaculture. For example, El-Houseiny et al. [11] showed that fish fed with synthesized AgNPs utilizing *M*. *oleifera* extract exhibited a substantial decrease in death rates, no cytotoxic effects, and protection against the genotoxic, splenotoxic, hepatotoxic, nephrotoxic, and immunosuppressant effects of *A*. *hydrophila* in Nile tilapia [19,20], offering a potential alternative to conventional antibiotic treatments. Oral delivery of nano-vaccines has also demonstrated promise in combating *A*. *hydrophila* infections in fish [21,22]. Infectious diseases represent a major challenge in aquaculture, particularly in intensive fish farming.

Although the utilization of antibiotics to treat these illnesses has been widespread, their overuse has resulted in several environmental concerns, including the development of antibiotic-resistant bacteria. Green nanotechnology offers a more sustainable and effective approach to disease management in aquaculture. Therefore, the application of nanotechnology to control or inhibit bacterial pathogens in aquatic environments is crucial. We hypothesized that coated silver nanoparticles (CAgNPs) would exhibit greater antibacterial activity than NCAgNPs, leading to improved growth performance and blood health in tilapia. This study comprised two parts. First, the *in vitro* antibacterial activity of CAgNPs and non-coated silver nanoparticles (NCAgNPs) was evaluated against seven bacterial pathogens: *Staphylococcus aureus*, *Bacillus cereus*, *A. hydrophila*, *Escherichia coli*, *Salmonella enterica*, *Aeromonas sobria*, and *Listeria monocytogenes*. Second, the *in vivo* effects of CAgNPs and NCAgNPs on blood biochemistry, immune response, and redox status were investigated in Nile tilapia infected with *A. hydrophila*.

## Materials and Methods

### Ethical approval

The current experiment and aquaculture animal methods procedures were approved according to the guidance of the Research Ethical Committee (IACUC-2024) at King Abdulaziz University in Saudi Arabia’s Faculty of Sciences, Department of Biological Sciences.

### Synthesis of coated silver nanoparticles (CAgNPs)

CAgNPs were synthesized from silver nitrate (Sigma Aldrich, ID: 209139) using a method adapted from Ng and Zheng [23]. UV-Vis spectroscopy confirmed the successful synthesis of CAgNPs, with a characteristic peak shown at 420 nm. TEM (transmission electron microscopy) revealed that the CAgNPs were spherical in form with a medium size of 50–80 nm. The analysis of zeta potential indicated a charge on the net surface of –22 mV. Dynamic light scattering (DLS) analysis determined the size of the hydrodynamic CAgNPs to be 57 nm.

### In vitro study

The bacterial pathogens *S. aureus*, *L. monocytogenes*, *E*. *coli*, *S. enterica*, *B. cereus*, *A. sobria*, and *A. hydrophila* were obtained and identified from the Egyptian Center for Microbial Research at Ain Shams University.

The *in vitro* antimicrobial activity of CAgNPs and NCAgNPs was evaluated utilizing the method of agar well spread [24]. Bacterial cultures were produced on Tryptic Soy Agar (Italy, Milan, Biolif. TSA) at 37°C for 24 h and subsequently stored on Tryptic Soy Agar at 4°C. For the assay, bacterial species were sub-cultured in MHB (Mueller–Hinton broth) for 24 h at 37°C.

The suspension of bacteria was modified to a concentration of 1.1 × 10⁸ CFU/ml using a 0.5 McFarland standard. MHA (Mueller–Hinton agar) plates were swabbed with the bacterial suspension, and 6 mm diameter wells were created. The wells were filled with varying levels (400, 200, 100, and 0 µg/ml) of the test compounds. Following 24 h of growth at 37°C, measurements were made of the zones of inhibition in millimeters. All experiment groups were conducted in triplicate.

The antimicrobial impact of coated CAgNPs and NCAgNPs was assessed utilizing the standard method of broth dilution (CLSI M07-A8) by observing the microorganism’s growth in the agar broth. The minimum inhibitory concentration (MIC) was determined in BHI broth using serial two-fold dilutions of CAgNPs and NCAgNPs ranging from 0, 100, 200, and 400 µg/ml with a standardized bacterial concentration (10⁸ CFU/ml, equivalent to 0.5 McFarland’s standard). For 24 h, a control that contained solely colonized broth was left to develop at 37°C. MIC was defined as the lowest level of AgNPs that completely inhibited visible microbial growth. Turbidity in the pipes was visually assessed both after and before incubation to validate the determined MIC level.

### In vivo study

#### Animal and experimental management

Nile tilapia with an average initial body weight of 2.34 ± 0.013 gm was presented from the Aquaculture Research Hatchery of Fish. The research was carried out in compliance with the ARRIVE standards and authorized by King Abdulaziz University’s Faculty of Sciences Ethics Committee in Jeddah, Saudi Arabia.

Following a 15-day acclimation period, fish were divided at random among glass aquariums holding 75 L of tap water that had been dechlorinated. Before the experiment started, fish were given a basal ration without coated AgNPs. Every tank was equipped with an air stone connected to continuous aeration using a central air compressor. Daily maintenance included removing fish feces and replacing approximately 35% of the water. The photoperiod was kept at 12 h of light and 12 h of dark.

During the experiment, the following average water quality indicators were preserved: temperature, 29.12 ± 0.11°C; pH, 6.62 ± 0.04; dissolved oxygen, 6.91 ± 0.03 mg/l; ammonia, 0.11 ± 0.03 mg/l; nitrite, 0.14 ± 0.06 mg/l; and nitrate, 2.42 ± 0.04 mg/l. These parameters were evaluated in accordance with the guidelines described by Choudhary and Sharma [25].

#### Experimental design

Two hundred Nile tilapia were divided into 5 groups for treatment at random (*n* = 40 for every group). The group control was fed without supplementation (a basal diet). The 4 groups’ treatment received the basal diet addition with either 200 mg/kg or 100 mg/kg of coated AgNPs (CAgNPs100 and CAgNPs200, respectively) or 100 mg/ kg or 200 mg/kg of non-coated AgNPs (NCAgNPs 200 and NCAgNPs 100, respectively) [11].

**Figure 1.**
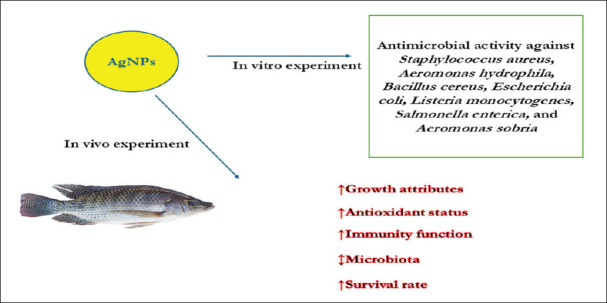
The simple diagram of experimental study.

The dietary ingredients were thoroughly mixed, kept at 4°C until needed, and then left to air dry for a full day at ambient temperature. The NRC was used to produce the basal diet [26] guidelines (Table 1). Fish were eaten three times every day at an amount of 5% of their total body weight. The feed ration for each replicate was modified biweekly based on differences in the total biomass of the fish. Figure 1 shows a simple diagram of the experimental study.

#### Growth indices and whole-body composition

Fish in each group were weighed biweekly to monitor changes in body weight, and feed intake was registered throughout the 80-day study. Feed conversion ratio (FCR), protein efficiency ratio (PER), specific growth rate (SGR), and survival rate (SR) were measured according to the methods described and used by Eldessouki et al. [27].

BWG (Body weight gain, gm) = FBW–IBW.

SGR (%/day) = 100 (Ln FWLn IW) / Period of study (days)

FCR = TFI / BWG

Weight gain (WG, %) = 100 (BWG / IBW

SGR (% d^–1^) = [ln final weight ln initial weight] × 100 / time (d).

SR (%) = 100 × (Number of survived fish / Initial number of fish stocked)

In conclusion of the feeding study, five fish from every group were randomly taken for whole-body composition analysis. CP (crude protein (*N* × 6.25)) was evaluated utilizing a Kjeldahl Distillation Unit (UDK 129, Italy), ash composition was evaluated utilizing a Muffle Furnace (Thermo Scientific, USA), and moisture content was measured using an oven convection (JSON-100, Korea). The crude lipid composition was evaluated utilizing a Soxhlet extractor. All analyses were performed according to standard procedures [28].

**Table 1.** Ingredients and nutrient contents of basal diet of growing *O. niloticus*.

Items	gm/Kg
Ingredients	
Yellow corn	210
Soybean meal	200
Wheat bran	150
Fish meal	150
Corn gluten meal	130
Extracted rice bran solvent	110
Soybean oil	30
^a^Vitamin premix	10
^b^Mineral premix	10
Analyzed composition (gmkg–1) as fed basis)	
^c^Crude protein (N × 6.25)	320.8
^d^Crude lipids	45.8
Crude fiber	42.3
Ash	73.3
Nitrogen free extract (NFE)	517.8
Gross energy (MJ per 100 gm)	18.21

^a^Composition of vitamin premix kg^–1^; vitamin A, 8,000,000 IU; vitamin D3, 2,000,000 IU; vitamin E, 7000 mg; vitamin K3, 1500 mg; vitamin B1, 700 mg; vitamin B2, 3500 mg; vitamin B6, 1000 mg; vitamin B12, 7 mg; biotin, 50 mg; folic acid, 700 mg; nicotinic, 20,000 mg; pantothenic acid, 7000 mg.

^b^Composition of mineral premix kg^–1^; manganese, 53 gm; zinc, 40 gm; iron, 20 gm; copper, 2.7 gm; iodine, 0.34 gm; selenium, 70 mg; cobalt, 70 mg, and calcium carbonate as carrier up to 1.0 Kg.

^c^NFE = 100 –(crude protein + Crude lipids + ash + crude fiber).

^d^Calculated using the factors 23.4 kJgm–1, 39.2 kJgm–1, and 17.2 kJgm–1 protein, fat and carbohydrate, respectively.

#### Plasma antioxidant and immunity analysis

At the end of the feeding trial (80 days), blood samples for testing were collected from the caudal vein of five fish per treatment group after anesthetization with MS-222 (tricaine methanesulfonate, 50–75 mg/l), following the style described by Katz et al. [29]. Blood was collected utilizing sterile, heparinized syringes and transferred to sterile pipes. Plasma was then disconnected by a centrifugation system at 3,000 rpm for 20 min and kept at –20°C.

Total antioxidant capacity (TAC), glutathione peroxidase (GPX), superoxide dismutase (SOD), malondialdehyde (MDA), and catalase (CAT) levels were measured utilizing commercially available ELISA kits (BioDiagnostic Company, Giza, Egypt): TAC-2513, GPX-2524, SOD-2521, MDA-2529, and CA2517, respectively. The assays were performed following established protocols of Young [30], Nishikimi et al. [31], Koracevic et al. [32], Huang et al. [33], and Kei [34] for assessing the TAC, SOD, GPX, CAT, and MDA, respectively.

Plasma IgG (MBS2800419), IgA (MBS034250), and IgM (MBS700823) values were measured using commercial ELISA kits by the manufacturer’s instructions. The IgG, IgA, and IgM ELISA kits had 9.375–600 ng/ml, 25–800 ug/ ml, and 12.5–800 ng/ml, respectively. All immunological ELISA kits were provided by the MyBioSource Company (San Diego, CA). Lysozyme impact was evaluated utilizing a turbidity-based assay [35]. The lysozyme kit had a minimum sensitivity of 75 pg/ml and a detection rate of 125–8,000 pg/ml.

#### Determination of intestinal bacteria

For microbiological examination, fresh digesta tests were aseptically taken from the posterior stomach. Sterile peptone as saline solution was used to serially dilute the digesta isolates (8.5 gm/l NaCl, 1 gm/l peptone) to determine TBC (total bacterial count, nutrient agar, and pour plate technique, 37°C for 24 h), coliform count, *E*. *coli* count (utilizing eosin methylene blue and the spread plate method, 44.5°C for 24 h), and *Aeromonas* spp. count utilizing the pour plate method with agar media selective, as described by [10,15,27]. *Aeromonas* spp. identification included assessing colony morphology, hemolysis on 5% sheep blood agar, oxidase testing, Gram staining, and profiling of phenotypic utilizing API 20 Strep and API 20 E (BioMerieux, France) kits. Every microbiological analysis was carried out twice, and the mean of the log10-transformed counts was used for statistical analysis. Following a 24 h incubation period at 37°C, the total bacterial counts were counted using the nutritional agar and pour plate method.

### Bacterial challenge

After 80 days, 20 fish from every therapy group were independently defied intraperitoneally with *A*. *hydrophila* at the measured LD50 dose. The fish were injected with 0.1 ml of harmful *A*. *hydrophila* (1.3 × 10⁸ CFU/ml) intraperitoneally [11]. The *A*. *hydrophila* strain was provided by King Abdulaziz University’s Faculty of Sciences’ Sustainable Agriculture Production Research Group in Saudi Arabia. The cumulative mortality rate was calculated after 14 days and evaluated using the following formula: RPS (relative percentage survival %) = [number of surviving fish after challenge/number of fish injected with bacteria] × 100.

### Statistical analysis

The consistency and normality of variance were evaluated using the Shapiro–Wilk and Levene’s tests, respectively. Statistical analysis was performed using SPSS 21 (Chicago, IL). One-way ANOVA was utilized to analyze the data, and Tukey’s multiple-range test was utilized for post hoc comparisons. The data are presented as the mean ± SEM. Statistical significance was set at *p* < 0.05.

## Results

### Characterization of AgNPs

The composition of AgNPs was confirmed by a color difference in the solution of reaction to reddish-brown. UV spectrophotometry analysis of AgNPs synthesized using *Bacillus pseudomycoides* filtrate (BPF) showed a strong absorbance band around 420 nm, referring to the existence of colloidal AgNPs with surface plasmon resonance peaks. TEM analysis further confirmed the formation of spherical AgNPs with a particle size in the 20–60 nm range, showing uniform size distribution without aggregation. The small particle size was attributed to the high level of bioactive elements in the BPF. Additionally, the AgNPs were coated with poly(lactic-co-glycolic acid) (PLGA) for further characterization. The reduction of AgNO₃ to AgNPs by *B*. *pseudomycoides* filtrate was evident from the color change and UV–Vis spectroscopy analysis, which showed a peak at 420 nm, indicating the formation of AgNPs.

### In vitro antibacterial activity of AgNPs

AgNPs and CAgNPs demonstrated significant antibacterial activity toward a range of harmful bacteria, including *S. aureus, L. monocytogenes, B. cereus, E. coli, A. hydrophila*, and *S. enterica*. The inhibition zones (IZ) produced by AgNPs ranged from 36.33 ± 0.88 to 9.33 ± 0.28 mm, significantly larger than the control group (22.0 ± 0.58 to 11.0 ± 0.18 mm).

Against the fish pathogen *A*. *hydrophila*, both AgNPs and coated AgNPs exhibited strong antibacterial activity, with IZ ranging from 10.0 ± 0.58 to 35.33 ± 0.88 mm (Table 2). Notably, higher concentrations of both uncoated (NCAgNPs) and CAgNPs AgNPs (specifically 100, 200, and 400 mg) resulted in greater inhibition of *A*. *hydrophila* compared to ampicillin (20.0 ± 0.58 mm). Coated AgNPs at 200 µg/ml were particularly effective, displaying the largest IZ (30.58 ± 0.85 to 36.33 ± 0.88 mm) across all tested pathogens. This was considerably greater than the IZ observed for penicillin (18.33 ± 0.13 mm) and ampicillin (22.0 ± 0.58 mm) under the same conditions. The MIC for both AgNPs and coated AgNPs toward the bacteria tested ranged from 100 to 200 µg/ ml (Table 3).

### Growth performance attributes

The various concentrations of NC-AgNPs and C-AgNPs demonstrated improvements in evaluated feed efficiency and growth performance traits (SGR, FI, FW, and DWG) in tilapia fish (Table 4). The highest PER and FCR were presented in the C-AgNPs100, NC-AgNPs200, and C-AgNPs200 groups. No significant variations in survival rate were conducted among all experimental groups (*p* > 0.05). The NC-AgNPs200, C-AgNPs100, and C-AgNPs200 groups exhibited the highest survival rates, ranging from 96% to 98%.

**Table 2.** Inhibition zone (mm) of NC-AgNP or C-AgNP levels against common bacterial strains *(in vitro*).

Treatment		Gram positive (mm)			Gram negative (mm)	
	*L. monocytogenes*	*S. aureus*	*B. cereus*	*S. enterica*	*A. hydrophila*	*E. coli*
Penicillin (PRA)	15.0 ± 0.53c	17.33 ± 0.02c	18.33 ± 0.13c	11.0 ± 0.18c	12.0 ± 0.28c	13.0 ± 0.18c
Ampicillin (PRA)	13.0 ± 0.58c	14.83 ± 0.0c	14.33 ± 0.33c	22.0 ± 0.58c	20.0 ± 0.58c	18.0 ± 0.58c
NC-AgNPs 100	10.33 ± 0.33d	9.67 ± 0.33d	10.67 ± 0.61d	11.0 ± 0.58e	10.0 ± 0.58e	9.33 ± 0.28e
NC-AgNPs 200	13.33 ± 0.33c	15.0 ± 0.38c	15.33 ± 0.13c	15.33 ± 0.67d	16.0 ± 0.58d	17.0 ± 0.58cd
NC-AgNPs 400	26.0 ± 0.58b	27.0 ± 0.58b	25.33 ± 0.18b	27.0 ± 0.25b	26.67 ± 0.18b	27.0 ± 0.38b
C-AgNP100	15.33 ± 0.28c	15.0 ± 0.58c	14.33 ± 0.85c	16.0 ± 0.51d	16.67 ± 0.67d	14.67 ± 0.33d
C-AgNP200	26.0 ± 0.58b	24.33 ± 0.81b	27.0 ± 0.58b	26.67 ± 0.18b	25.0 ± 1.52bc	27.67 ± 0.33b
C-AgNP400	30.67 ± 0.28a	30.0 ± 0.58a	32.67 ± 0.81a	34.33 ± 1.20a	35.33 ± 0.88a	36.33 ± 0.18a
SEM	0.52	0.48	0.57	0.63	0.71	0.52
*p*-value	0.0001	0.0001	0.0001	0.0001	0.0001	0.0001

Values within the same row having different superscripts are significantly different (*p* < 0.05). Values are expressed as mean ± SE.

NC-AgNPs = non-coated silver nanoparticle, C-AgNPs = coated silver nanoparticles; PRA: The reference standards used were ampicillin (10 mg/disc) for Gram-negative and Penicillin G (disc loaded with 10 units) for Gram-positive bacteria.

**Table 3.** Minimum inhibition concentration (µg) of AgNPs or CAgNPs against pathogenic bacterial strains *(in vitro*).

Pathogenic bacteria	Non-coated AgNPs (µg/ml)	Coated AgNPs (µg/ml)
*L. monocytogenes*	200 ± 0.56	100 ± 0.33
*S. aureus*	200 ± 0.34	100 ± 0.37
*B. cereus*	200 ± 0.57	100 ± 0.43
*S. enterica*	200 ± 0.24	150 ± 0.46
*E. coli*	200 ± 0.35	150 ± 0.45
*A. hydrophilia*	200 ± 0.53	150 ± 0.65

AgNPs = Silver nanoparticles, CAgNPs = coated silver nanoparticles.

### Effect of coated AgNPs on whole-body composition

**Figure 2.**
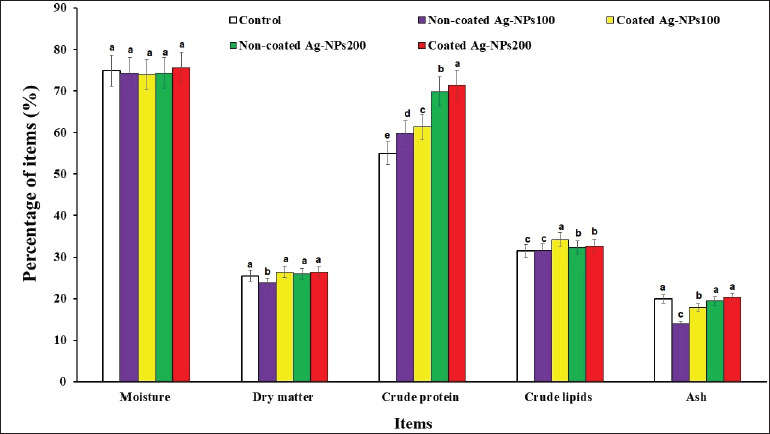
Effect of dietary NC-AgNPs or C-AgNPs levels on whole-body composition (% wet weight basis) of Nile tilapia fish. The first group was fed basal diet (control group). Fish were fed basal diets supplemented with 100 mg of CAgNPs (CAgNPs100), 200 mg of CAgNPs (CAgNPs200), 100 mg of NCAgNPs (NCAgNPs100), or 200 mg of NCAgNPs (NCAgNPs200) per kg of diet, respectively. a–c Values within the same row having different superscripts are significantly different (*p* < 0.05).

The impact of various types and doses of AgNPs on the whole-body components of tilapia fish is illustrated in Figure 2. No significant variations were observed in moisture value across all treatments. The non-coated AgNPs100 group showed the lowest dry matter content compared to the other treatments (*p* < 0.05). Furthermore, the highest crude protein rates were found in the coated AgNPs200 group, followed by the non-coated AgNPs200 group. Overall, all treated groups exhibited higher crude protein levels than the control group (*p* < 0.05). The coated AgNPs100 group had the highest crude lipid content, while both coated and non-coated AgNPs200 groups showed higher crude lipid levels than the group control and non-coated AgNPs100 groups (*p* < 0.05). Regarding ash content, the AgNPs200 groups (coated and non-coated) and the group control had the highest ash content, while the non-coated AgNPs100 treatment had the lowest (*p* < 0.05).

### Effect of C-AgNPs on immunological parameters of O. niloticus

**Figure 3.**
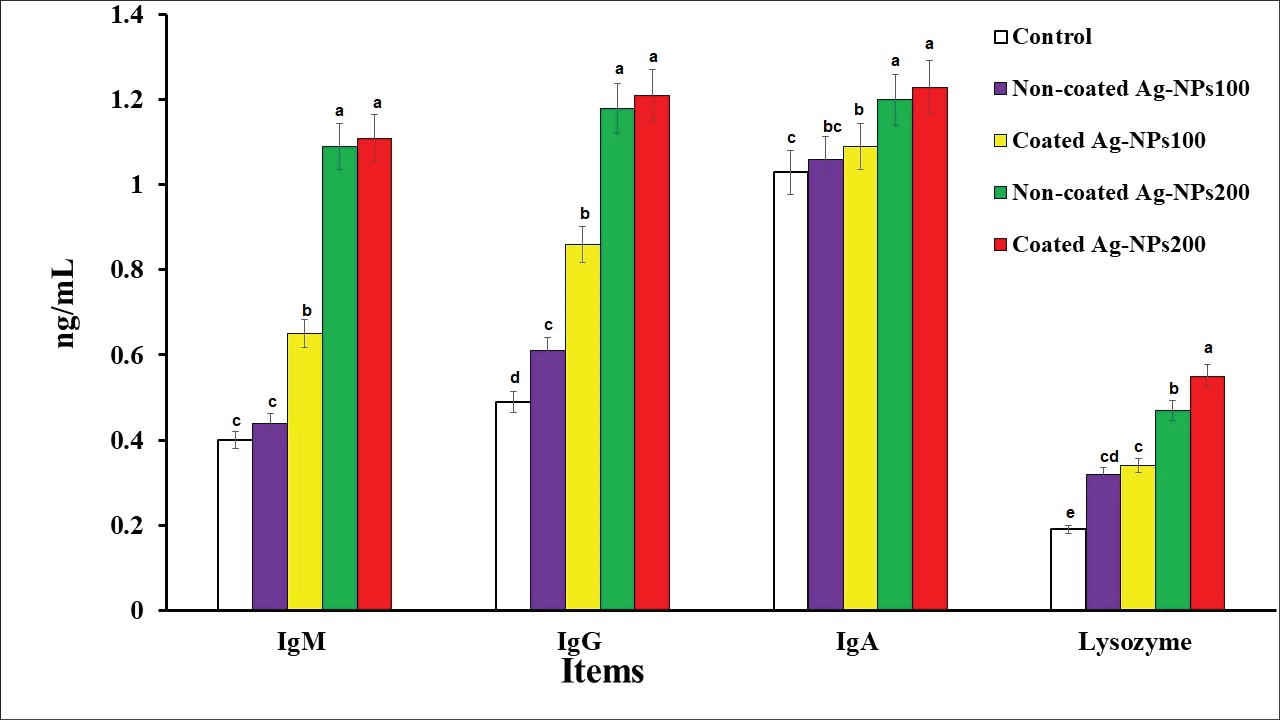
Effect of dietary NC-AgNPs or C-AgNPs levels on immunological parameters of Nile tilapia fish. The first group was fed basal diet (control group). Fish were fed basal diets supplemented with 100 mg of CAgNPs (CAgNPs100), 200 mg of CAgNPs (CAgNPs200), 100 mg of NCAgNPs (NCAgNPs100), or 200 mg of NCAgNPs (NCAgNPs200) per kg of diet, respectively. a–dValues within the same row having different superscripts are significantly different (*p* < 0.05).

Figure 3 presents the effects of various types and doses of AgNPs on the immunological parameters in tilapia fish. Notably, the non-coated AgNPs200 and coated AgNPs200 groups showed significantly higher values of IgM, IgG, and IgA compared to the other treatment groups (*p* < 0.05). The coated AgNPs100 group demonstrated intermediate values for all immune markers. There were no significant differences in IgM, lysozyme level, or IgA value between the control treatment and the non-coated AgNPs100 group (*p* > 0.05). However, lysozyme activity was particularly higher in the coated AgNPs200 treatment, followed by the non-coated AgNPs200 treatment (*p* < 0.05). The coated AgNPs100 group displayed intermediate lysozyme activity. These findings indicate that both coated and noncoated AgNPs at a level of 200 ng/ml remarkably boosted the immune responses in tilapia fish.

### Effect of C-AgNPs on oxidative status of O. niloticus

**Figure 4.**
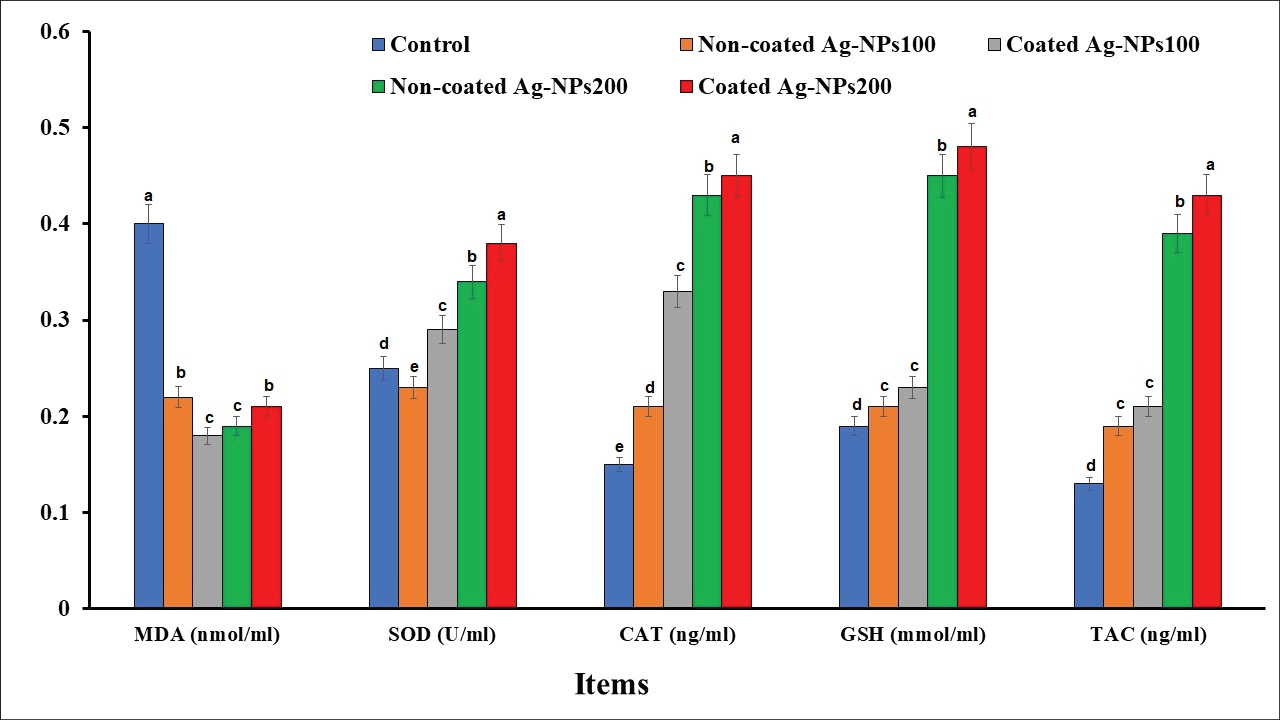
Effect of dietary NC-AgNPs or C-AgNPs levels on oxidative status of Nile tilapia fish (*O*. *niloticus*). The first group was fed basal diet (control group). Fish were fed basal diets supplemented with 100 mg of CAgNPs (CAgNPs100), 200 mg of CAgNPs (CAgNPs200), 100 mg of NCAgNPs (NCAgNPs100), or 200 mg of NCAgNPs (NCAgNPs200) per kg of diet, respectively. a–d, Values within the same row having different superscripts are significantly different (*p* < 0.05).

Figure 4 illustrates the impact of various AgNP types on oxidative stress markers in tilapia fish, including MDA (lipid peroxidation level) and antioxidant enzyme levels (SOD, CAT, GSH, and TAC). The lowest MDA values were shown in the coated AgNPs100 and non-coated AgNPs200 groups (*p* < 0.05) compared to the control and other treatments. Conversely, the non-coated AgNPs100 and coated AgNPs200 groups had higher MDA levels than other treated groups, though still lower than the control treatment (*p* < 0.05).

**Table 4.** Growth performance of Nile tilapia fed with diets containing different concentrations of NC-AgNPs or C-AgNPs.

Parameter	CNT	C-AgNP_100_	C-AgNP_200_	NC-AgNP_100_	NC-AgNP_200_	Linear	Quadratic
IW (gm/fish)	2.57 ± 0.34	2.57 ± 0.56	2.55 ± 0.34	2.56 ± 0.23	2.57 ± 0.33	0.632	1.002
FW (gm/fish)	23.45 ± 0.37d	24.45 ± 0.57c	24.67 ± 0.37c	25.56 ± 0.28b	26.45 ± 0.33a	0.126	<0.001
DWG (mg/day)	233.45 ± 6.56d	236.57 ± 4.45c	237.48 ± 4.67c	241.48 ± 5.58b	248.57 ± 4.65a	0.164	<0.001
SGR (%)	3.88 ± 0.11c	4.56 ± 0.35b	4.68 ± 0.38b	4.78 ± 0.42a	4.89 ± 0.22a	0.095	<0.001
FI (mg/day)	427 ± 6.68e	466 ± 6.34d	487 ± 5.78c	489 ± 5.87b	495 ± 6.69a	0.597	<0.001
FCR (gm/gm)	1.85 ± 0.11	1.82 ± 0.09	1.81 ± 0.05	1.78 ± 0.18	1.75 ± 0.17	0.024	0.012
PER (gm/gm)	1.72 ± 0.14	1.76 ± 0.16	1.79 ± 0.15	1.83 ± 0.13	1.86 ± 0.17	0.026	0.013
Survival rate (%)	90.35 ± 0.23	94.45 ± 0.38	96.45 ± 0.26	96.89 ± 0.36	97.78 ± 0.45	0.735	0.393

CNT: Control; a-d, Values within the same row having different superscripts are significantly different (*p* < 0.05). Data were presented as the mean ± standard error.

IW = initial wet weight, FW = final wet weight, DWG = daily weight gain, SGR = specific growth rate, FI = feed intake, FCR = food conversion ratio, PER = protein efficiency ratio, NC-AgNPs = non-coated silver nanoparticles, CAgNPs = coated silver nanoparticles, CNT = control.

Regarding antioxidant markers, the coated AgNPs200 treatment exhibited the highest values of SOD, GSH, CAT, and TAC compared to the other treatments (*p* < 0.05). The non-coated AgNPs200 group showed elevated antioxidant enzyme levels as well, surpassing both the control group and the 100 ng treatments, whether coated or non-coated. The values of GSH and TAC in the non-coated AgNPs100 and coated AgNPs100 groups were similar (*p* > 0.05), but both were remarkably higher than those in the group of control (*p* < 0.05). Overall, the administration of AgNPs, whether coated or non-coated, contributed to enhanced antioxidant capacity in tilapia fish while reducing lipid peroxidation.

## Intestinal bacteria changes

The data presented in Table 5 illustrate the variations in the amounts of bacteria in intestinal groups (TBC, *E*. *coli*, coliform group*,* and *A*. *hydrophila*) in fish (Nile tilapia) fed varying concentrations of NC-AgNPs and C-AgNPs when the experiment was ended. The TBC was reduced in the NC-AgNPs and C-AgNPs fed treatments at all levels linked to the group control in fish samples from the intestine. Additionally, *Aeromonas* spp., *coliform* number, and *E. coli* counts were reduced in the NC-AgNPs- and C-AgNPs-treated treatments compared to the group control in the period of feeding.

**Table 5.** Total bacterial count, Coliform, *E*. *coli* and *A*. *hydrophila* count of Nile tilapia fish fed diets containing 0.0, 100, 200 CAgNPs and 100, 200 AgNPs µg kg^-1^ diet.

Parameters			Experimental diets				*p* value
	CNT	C-AgNP_100_	C-AgNP_200_	NC-AgNP_100_	NC-AgNP_200_	Linear	Quadratic
TBC*	6.68 ± 0.02^a^	4.81 ± 0.05^b^	4.78 ± 0.02^b^	4.59 ± 0.04^bc^	4.84 ± 0.01^bc^	0.0001	0.0001
Coliform	5.35 ± 0.02^a^	4.20 ± 0.02^b^	4.17 ± 0.02^b^	4.04 ± 0.02^bc^	3.82 ± 0.11^c^	0.0001	0.235
*E. coli*	2.17 ± 0.04^a^	1.04 ± 0.02^b^	0.98 ± 0.04^c^	0.90 ± 0.03^c^	0.72 ± 0.07^c^	0.0001	0.0001
*A. hydrophila*	2.14 ± 0.03^a^	1.02 ± 0.01^b^	0.97 ± 0.05^b^	0.90 ± 0.03^c^	0.77 ± 0.04^c^	0.0001	0.0001

^*^TBC: total bacterial count; a-d, Values within the same row having different superscripts are significantly different (*p* < 0.05). Data were presented as the mean ± SE.

NC-AgNPs = non-coated silver nanoparticles, CAgNPs = coated silver nanoparticles, CNT = control.

**Figure 5.**
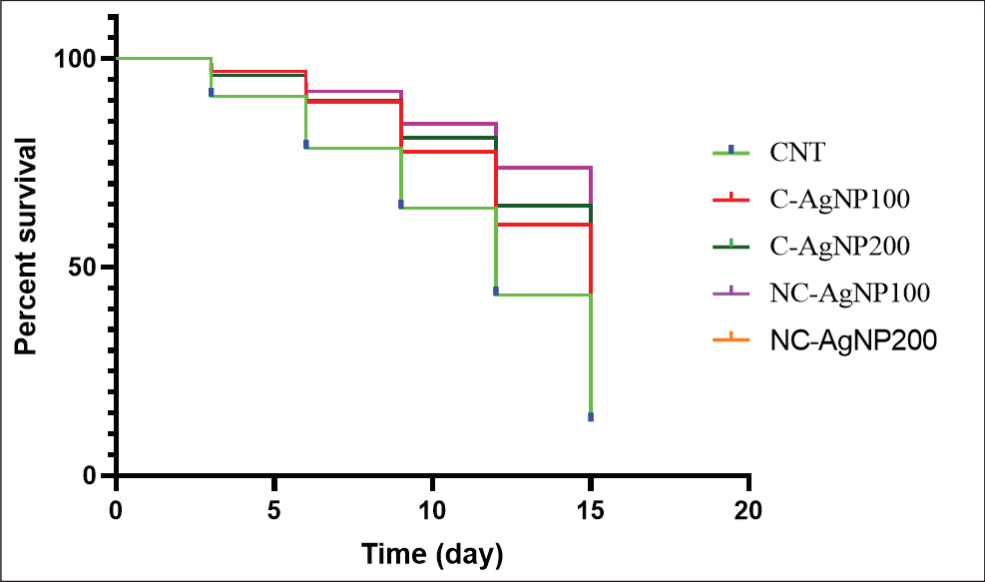
Effect of dietary NC-AgNPs or C-AgNPs levels on the survival rate of Nile tilapia fish (*O*. *niloticus*) after infection with *Aeromonas hydrophila*.

## Bacteria challenge

Figure 5 demonstrates that treatment with CAgNPs at both concentrations (100 and 200) led to significantly higher survival rates compared to the other treatments.

## Discussion

This study reveals that silver-coated nanoparticles (AgNPs) are more effective than tested antibiotics in mitigating *A*. *hydrophila* virulence. These findings suggest that AgNPs hold promise for developing targeted interventions against *A*. *hydrophila* infections in aquaculture. Many researchers have established the antibacterial impacts of AgNPs toward a range of fish pathogens [1,11,12] and explored their toxic effects in certain fish species. Our work specifically demonstrates their superior performance against *A*. *hydrophila* virulence. Further investigation is warranted to optimize AgNP delivery and minimize potential toxicity while maximizing their therapeutic benefits. Considering the influence of environmental factors (e.g., temperature, pH, salinity) on AgNP stability and efficacy is crucial for developing effective disease control strategies. The potential of AgNPs as eco-friendly and cost-effective alternatives to traditional antibiotics offers a significant advantage for sustainable aquaculture practices.

AgNPs are effective in controlling microbial growth and reducing water contamination [36]. Coated AgNPs are applied for topical drug delivery and nanomedical treatments [37,38]. AgNPs are excellent substitutes for antimicrobial factors [37]. The effect of silver NPs as antifungal, anti-inflammatory, antiviral, antibacterial, and anticancer factors has been demonstrated by many researchers [37,39].

Growth performance is a fundamental aspect of aquaculture, as it directly impacts the productivity and economic viability of fish farming. In this context, growth performance encompasses factors such as weight gain, feed conversion efficiency, and overall health, which are crucial for assessing the success of farming practices. Recent research by Popoola et al. [40] highlighted the positive impacts of AgNPs on growth performance in rohu (*Labeo rohita*). Specifically, their study found that feeding rohu with 15 µg of AgNPs significantly enhanced growth indices, including weight gain and feed efficiency [40], indicating that AgNPs could potentially serve as a beneficial addition in fish diets for enhancing overall productivity and growth [40].

In contrast, another research by Aly et al. [41] conducted the effects of AgNPs on tilapia (*Oreochromis* spp.) and concluded that AgNPs did not cause any significant enhancement in growth performance. This finding suggests that the impact of AgNPs on fish growth might vary depending on species, dosage, and experimental conditions. The contrasting results from these two studies underline the need for further research to better understand how different types and concentrations of AgNPs affect various fish species and whether their potential benefits can be optimized for specific aquaculture applications. Thus, while AgNPs may hold promise for enhancing growth in certain species, more comprehensive studies are required to determine their overall efficacy in fish farming.

A study by Chang et al. [42] found that in pools with hyperfeed, the spread of *Aeromonas* spp. bacteria was raised. This raises concerns since leftover feed can cause rapid growth of virulent bacteria (*A*. *hydrophila)*, causing fish mortality when its density reaches 108 CFU/ml. Our results showed that the addition of AgNPs mixed with feed caused enhanced growth performance and protected the diet moreover, prevented species of *Aeromonas* from growing*.* In particular, the nutrient that allowed the bacteria to succeed in fish cultivation pools and generate infection occurrences is unspecified. Additionally, it is unclear whether nutrients explicitly provide for *A*. *hydrophila’s* infection. AgNP biosynthesis by the green method did not ignore the poisonous purposes at specific doses that have been informed [11].

The nanomaterial under consideration could be utilized in water management to manage or shrink disease outbreaks caused by bacterial fish pathogens. In a study, fish injured with *A. hydrophila* and handled with 0.8 mg/l of AgNPs did not exhibit any adverse health effects. Silica nanoparticles (3 mg/kg diet) have been found to improve the lipid content in tilapia fish muscle [43].

These results are harmonized with previous research that has investigated the impact of nanoparticles on intestinal health. Recognizing the immune classification and developing new tactics for administering immunity in aquaculture are crucial for addressing disease involvement in tilapia farming. Recently, in alignment with our data [40], when feeding rohu (*Labeo rohita*) with coated AgNPs, 10–20 µgKg^–1^ significantly improved immunity and significantly reduced inflammation markers, such as TGF-β, COX-2, and IL-8. The study by Saad et al. [44] suggested that CAgNPs are effective against microorganisms that are resistant to drugs such as *A*. *hydrophila* and *P*. *aeruginosa, species* commonly associated with aquaculture. Our study reported that dietary supplementation with AgNPs can significantly improve the immune response in tilapia fish. These results suggest that the addition of AgNPs may have the potential to serve as an immunostimulant in aquaculture, possibly improving disease resistance and overall fish health.

Aquaculture practices can subject fish to a range of stressors, including environmental changes, handling, overcrowding, and disease outbreaks [2]. These stressors can disrupt the fish’s natural redox balance, which is the equilibrium between ROS (reactive oxygen species) and antioxidant defenses in the body. When this balance is disturbed, oxidative stress occurs, leading to an accumulation of ROS that can damage cellular components, such as lipids, proteins, and DNA [43]. Over time, this damage can impair the fish’s health, growth, and immune function, ultimately affecting their overall productivity in aquaculture systems [4]. As a result, managing oxidative stress is crucial in aquaculture to ensure fish health and optimize the efficiency of farming practices [22]. This oxidative stress manifests as elevated lipid peroxidation, as evidenced by increased MDA levels.

Concurrently, the impact of antioxidant enzymes, such as SOD, CAT, and GSH, can be significantly reduced, indicating impaired cellular homeostasis. Furthermore, oxidative stress has been shown to induce DNA damage in various cell types, including immune cells. In response to these challenges, fish employ both non-enzymatic and enzymatic antioxidant defense roles to mitigate oxidative stress and neutralize the excessive generation of free radicals [27]. Research by Bashar et al. [43] reported that dietary addition with silica nanoparticles can enhance antioxidant enzyme activity in tilapia fish, suggesting a potential immunomodulatory effect.

Iron nanoparticles can enhance the antioxidant capacity in fish (common carp), as demonstrated by Hussain et al. [45]. The oxidation level in fish can be evaluated by measuring lipid peroxidation and MDA levels, with CAT and SOD activities reflecting antioxidant responses [46]. Tilapia treated with C-AgNPs exhibited elevated SOD and CAT levels and reduced MDA levels. Lysosome activity was also decreased in fish given 10 µg/l of AGNPs in water [46]. These nanoparticles possess unique properties that make them effective in enhancing the function of enzymes that protect cells from oxidative stress. AgNPs can enhance antioxidant enzymes by increasing their stability and activity [47]. The nanoparticles can interact with the enzymes and create a protective environment, helping the enzymes maintain their structure and function under stressful conditions. Furthermore, C-AgNPs can act as scavengers of free radicals, highly reactive molecules that can damage cells and contribute to various diseases. Additionally, AgNPs possess anti-inflammatory properties, which can further reduce inflammation in the body by supporting antioxidant status [47]. Inflammation and oxidative stress are closely intertwined, and by mitigating inflammation, AgNPs can indirectly support the function of antioxidant enzymes. Coated AgNPs contribute significantly to improved antioxidant enzyme activity through several mechanisms, including free radical scavenging, enhancing enzyme stability, and attenuating inflammation. These properties make C-AgNPs a promising candidate for therapeutic applications in combating oxidative stress-related diseases.

The total bacteria count in the water diminished in response to the addition of 10 µg/l [48]. The same authors found that the survival rate also improved at low AgNP concentrations but decreased at higher exposure levels [48]. The antimicrobial activity of CAgNPs was also evidenced by several authors [12,46]. Fish fed the 15 µg of AgNPs/kg diet had significantly higher post-challenge survival rates (90%), suggesting that feed inclusion of AgNP at 10 and 15 µg/kg diets improves growth, immune response, and health toward *A*. *hydrophila* [40]. Additional findings are needed to determine the protective impact of AgNPs and the required recovery time for their safe use as antibacterial agents in different fish species. AgNPs have unique properties that make them effective in combating harmful bacteria in the gut. When AgNPs come into contact with pathogenic bacteria, they have the ability to damage the membrane of bacterial cells, which results in cell death. This mechanism of action makes AgNP a promising method in the fight against intestinal spread caused by harmful bacteria [49]. Researchers have found that AgNPs can impact the growth of pathogenic bacteria, such as *A*. *hydrophila* [50]. By inhibiting the growth of these bacteria, AgNPs can help prevent infections and promote gut health.

AgNPs have demonstrated minimal toxicity to human cells, positioning them as a safe and effective solution for targeting harmful bacteria in the gastrointestinal tract. Their application to reduce pathogenic intestinal bacteria holds considerable potential for enhancing gut health and preventing infections. However, additional research is required to get a more profound understanding of the mechanisms by which AgNPs function and their broader potential applications in combating harmful gut bacteria. This study has some limitations. Future research should incorporate histological analysis of internal organs (e.g., intestines, liver) and assess the possible toxicity of the AgNP, especially given their use as antibacterial agents and growth promoters.

## Conclusion

This study demonstrated the *in vitro* antimicrobial efficacy of silver-coated nanoparticles toward bacterial pathogens that pose a significant threat to tilapia aquaculture. Furthermore, *in vivo* experiments demonstrated that coated AgNPs improved growth performance and overall tilapia health by bolstering the immune response (IgM, IgG, IgA, and lysozyme activity), enhancing antioxidant capacity (CAT, TAC, and GSH), and reducing lipid peroxidation and pathogenic microbiota in the gastrointestinal tract. These beneficial effects resulted in increased survival rates following *A*. *hydrophila* infection. This approach offers a sustainable and effective alternative to conventional antibiotics and synthetic treatments for managing bacterial diseases in aquaculture. However, this study has limitations that warrant further investigation. First, the long-term effects of AgNPs on fish health and the environment were not fully assessed. The potential for bioaccumulation in fish tissues and the impact on the wider aquatic ecosystem remain unclear. Future research should prioritize evaluating the chronic toxicity of AgNPs and their long-term effects on non-target aquatic organisms. Second, while this study examined key immune and antioxidant parameters, additional investigations, such as histopathological analysis of internal organs (e.g., liver, kidney, and intestine), would offer a more thorough comprehension of the physiological responses to AgNPs exposure. Furthermore, the impact of AgNPs on the tilapia microbiome, crucial for gut health and microbial balance, was not evaluated. Integrating *16S rRNA* gene sequencing in future studies would provide valuable insights into changes in gut microbiota composition following nanoparticle treatment. Future research directions include optimizing the dosage and coating strategies for AgNPs to maximize efficacy while minimizing potential risks. Exploring synergistic effects between AgNPs and other environmentally friendly treatments, such as plant-derived compounds or probiotics, could lead to more sustainable disease management strategies in aquaculture. Finally, addressing the regulatory and ethical considerations surrounding nanomaterial use in aquaculture will be essential for ensuring their safe and responsible application.

## List of abbreviations

AgNPs, silver nanoparticles

CAgNPs, coated silver nanoparticles

CAT, catalase

CNT, control

DWG, daily weight gain

FCR, feed conversion ratio

FI, feed intake

FW, final wet weight

GPX, glutathione peroxidase

IW, initial wet weight

MDA, malondialdehyde

NCAgNPs, noncoated silver nanoparticles

PER, protein efficiency ratio

SOD, superoxide dismutase

specific growth rate

SR, survival rate

TAC, total antioxidant status

TBC, total bacterial count.
